# Investigating the Role for IL-21 in Rabies Virus Vaccine-induced Immunity

**DOI:** 10.1371/journal.pntd.0002129

**Published:** 2013-03-14

**Authors:** Corin L. Dorfmeier, Evgeni P. Tzvetkov, Anthony Gatt, James P. McGettigan

**Affiliations:** 1 Department of Microbiology and Immunology, Jefferson Medical College, Thomas Jefferson University, Philadelphia, Pennsylvania, United States of America; 2 Jefferson Vaccine Center, Jefferson Medical College, Thomas Jefferson University, Philadelphia, Pennsylvania, United States of America; 3 Kimmel Cancer Center, Jefferson Medical College, Thomas Jefferson University, Philadelphia, Pennsylvania, United States of America; Institut Pasteur de Tunis, Tunisia

## Abstract

Over two-thirds of the world's population lives in regions where rabies is endemic, resulting in over 15 million people receiving multi-dose post-exposure prophylaxis (PEP) and over 55,000 deaths per year globally. A major goal in rabies virus (RABV) research is to develop a single-dose PEP that would simplify vaccination protocols, reduce costs associated with RABV prevention, and save lives. Protection against RABV infections requires virus neutralizing antibodies; however, factors influencing the development of protective RABV-specific B cell responses remain to be elucidated. Here we used a mouse model of IL-21 receptor-deficiency (IL-21R−/−) to characterize the role for IL-21 in RABV vaccine-induced immunity. IL-21R−/− mice immunized with a low dose of a live recombinant RABV-based vaccine (rRABV) produced only low levels of primary or secondary anti-RABV antibody response while wild-type mice developed potent anti-RABV antibodies. Furthermore, IL-21R−/− mice immunized with low-dose rRABV were only minimally protected against pathogenic RABV challenge, while all wild-type mice survived challenge, indicating that IL-21R signaling is required for antibody production in response to low-dose RABV-based vaccination. IL-21R−/− mice immunized with a higher dose of vaccine produced suboptimal anti-RABV primary antibody responses, but showed potent secondary antibodies and protection similar to wild-type mice upon challenge with pathogenic RABV, indicating that IL-21 is dispensable for secondary antibody responses to live RABV-based vaccines when a primary response develops. Furthermore, we show that IL-21 is dispensable for the generation of T_fh_ cells and memory B cells in the draining lymph nodes of immunized mice but is required for the detection of optimal GC B cells or plasma cells in the lymph node or bone marrow, respectively, in a vaccine dose-dependent manner. Collectively, our preliminary data show that IL-21 is critical for the development of optimal vaccine-induced primary but not secondary antibody responses against RABV infections.

## Author Summary

Over two-thirds of the world's population lives in regions where rabies is endemic, resulting in over 15 million people receiving post-exposure treatment. A person, disproportionately a child, dies of rabies every 20 minutes and the cost of rabies prevention exceeds $1 billion US dollars per year. The development of a single-dose human rabies vaccine would greatly reduce the burden of rabies globally by lowering the cost associated with rabies vaccination and saving lives. Understanding how B cells develop to produce protective virus neutralizing antibodies would greatly help to achieve the goal of developing a single-dose vaccine. In this report, we show that IL-21 is critical for the induction of primary vaccine-induced anti-RABV G antibody titers and that the effects of IL-21 are highly dependent on the dose of vaccine administered. In our model of rabies immunogenicity and protection, the lack of IL-21 receptor influenced the detection of B cells in germinal centers in lymph nodes or of plasma cells in bone marrow after immunization with low or high doses of vaccine, respectively. Overall, these preliminary results indicate that IL-21 has the potential to influence B cell development and functions in the context of rabies vaccine-induced immunity and protection.

## Introduction

RABV is a single-stranded negative sense RNA virus of the genus lyssavirus in the *Rhabdoviridae* family that kills approximately 55,000 people annually. Up to 60% of rabies cases are in children, making rabies the seventh most important infectious disease in terms of years lost [1]. In Africa, a person dies of rabies every 20 minutes [2]. In China, rabies became the leading cause of infectious disease mortality in 2006, which increased by more than 27% from 2005 [3]. In the United States, cases of rabies in wildlife are detected in virtually all states and Puerto Rico (Hawaii is considered rabies-free). Except for cattle and foxes, the incidence of rabies in domesticated or wildlife remained unchanged or significantly increased in the US in 2011 compared to the five-year average for each species [4], exemplifying the difficulty in containing zoonotic viral infections even in industrialized nations. The cost associated with rabies in the US, Africa and Asia is almost $1 billion annually [5], [6] contributing to the financial burden of global health care costs. Furthermore, rabies is a NIAID Category C Priority Pathogen, indicating rabies is an emerging infectious disease with the potential for mass dissemination and harm to people [7]. Together, rabies is considered a neglected global zoonotic infectious disease that disproportionately affects children and, therefore, understanding how B cells develop in response to experimental RABV-based vaccination may help to support efforts to develop a single-dose human rabies vaccine for use in both developing and industrialized countries.

A wide array of RABV variants exist, ranging from highly pathogenic strains to attenuated RABV vaccine strains such as the molecular clone SAD B19 [8]. Live attenuated RABV vaccine strains are highly immunogenic and potentially could serve as a single-dose human RABV vaccine to replace currently used multi-dose inactivated RABV-based vaccine regimens. Due to residual pathogenicity of these live virus strains, however, several “second-generation” RABV-based vaccines are under investigation in which entire genes are deleted from the RABV genome [9]–[12], or multiple pathogenic markers are genetically modified [13]. Data from these studies indicate that very safe and effective live RABV-based vaccine vectors can be generated. Despite extensive efforts to attenuate live RABV-based vaccine vectors for safety, little information is available on factors that influence the generation of effective antibodies in response to live RABV-based vaccines. Virus neutralizing antibodies (IgG but not IgM) directed against the RABV glycoprotein (G) are protective against pathogenic RABV strains [14], [15]. In the case of a replication-deficient RABV-based vaccine in which the matrix gene is deleted, VNAs are generated by T cell-independent and –dependent (extrafollicular and germinal center) mechanisms [16], suggesting multiple pathways of B cell activation and differentiation could be exploited to rationally design a single-dose RABV vaccine for use in both pre- and post-exposure settings. With respect to typical vaccine-induced antibody responses, APC-primed T cells most likely display an intermediate T_fh_ phenotype (i.e., “pre-T_fh_ cell”) characterized phenotypically as CD4^+^CXCR5^hi^PD1^lo^, which migrate the T and B cell border of secondary lymphoid organs and interact with their cognate antigen-primed B cells [17]. This T∶B cell interaction typically results in the T_fh_ cells producing optimal amounts of IL-21, and in the B cells differentiating into early short-lived extrafollicular antibody secreting cells or migrating into the follicles and forming GCs. With additional signals provided by T_fh_ cells in GCs, B cells mature and differentiate into long-lived plasma cells (PCs) secreting high affinity antibodies or into memory B cells. Due to the importance for PCs secreting high affinity antibodies and memory B cells in vaccine-induced immunity, the development of T_fh_ cells and CG B cells is critical for vaccine-induced protection against future exposures. In the context of RABV-specific vaccination in post-exposure settings, the rapid induction of extrafollicular B cell responses may also be critical to prevent infection of the CNS, especially in cases where treatment is delayed after exposure to a potentially infected animal. As such, understanding factors that generate short- and long-term anti-viral B cell responses will help design more efficacious RABV vaccines for use in humans.

Cytokines present at the time of antigen exposure influence T and B cell activation and GC formation and, therefore, also affect the outcome of vaccination. IL-21 [18], [19] is a type 1 cytokine that is a member of the common γ-chain receptor family, which also includes IL-2, IL-4, IL-7, IL-9, and IL-15. It is produced primarily by activated T_fh_ and Th17 cells and has pleiotropic effects throughout innate and adaptive immunity [reviewed in [20]]. The role for IL-21 in regulating T_fh_ and B cell functions was originally identified using model antigens [21]–[23]. In addition, the role for IL-21 in immunity and protection against helminth [24], viral [25], [26] and bacterial [22] infections has been studied. IL-21 is a key mediator for the control of persistent viral infections in mouse models of LCMV [27]–[29] and hepatitis B virus [30], or in humans infected with HIV [31]–[33] or HIV in combination with the 2009 H1N1 influenza virus vaccine [34]. However, the complexity and diversity of persistent infections makes it is difficult to pinpoint the effects of IL-21 on anti-viral B cells versus CD8^+^ T cells, both of which can contribute the control of many chronic infections [35]. Conversely, B cells play a critical role for clearance of most acute viral infections and for the efficacy of vaccines against most vaccine-preventable diseases. Clearance of RABV infections relies strictly on B cell-mediated effector functions, but not CD8^+^ T cells, for protection, making RABV infection an excellent mouse model to pinpoint the role for IL-21 in vaccine-induced immunity against RABV infections and potentially for other pathogens that rely solely on B cells for protection. In this report, our preliminary data indicate that IL-21 is critical for the development of effective vaccine-induced primary antibody responses against RABV infections by influencing GC B cells or PC generation in a vaccine dose-dependent manner, while also showing IL-21 is dispensable for RABV-specific secondary antibody responses when a primary antibody response develops.

## Materials and Methods

### Ethics statement

All animal work was reviewed and approved by the Institutional Animal Care and Use Committee (IACUC) of Jefferson Medical College, Thomas Jefferson University. Work was completed in accordance with international standards [Association for Assessment and Accreditation of Laboratory Animal Care (AAALAC)] and in compliance with Public Health Service Policy on Humane Care and Use of Laboratory Animals, The Guide for the Care and Use of Laboratory Animals of the National Institutes of Health (NIH).

### Viral vaccine, challenge rabies virus and mice

The construction of the live RABV-based vaccine (rRABV) used in this study was described elsewhere and was previously named SPBN [9], [36], [37]. This vaccine is a molecular clone derived from the attenuated SAD-B19 vaccine strain of RABV. Virus stocks were propagated on baby hamster kidney cells and then concentrated and purified over a 20% sucrose cushion. The challenge virus used was the pathogenic Challenge Virus Strain-N2c (CVS-N2c), which is a mouse-adapted sublclone of CVS-21 RABV [38]. CVS-N2c was initially propagated in neonatal mouse brains and then passaged once in-vitro on a neuroblastoma cell line (NA cells). The titer of CVS-N2c required to kill unvaccinated mice was determined experimentally by inoculating serial ten-fold dilutions into naïve immune-competent mice [39] i.m. and then observing mice daily for clinical neurological symptoms of rabies. The titer required to kill unvaccinated mice within 8 days post-infection, which is typical for CVS-N2c [9], [10], was determined to be 10^5^ focus forming units (ffu)/mouse. Cryopreserved embryos of mice deficient in the IL-21 receptor (B6;129-IL21r *^tm1Wjl^*/Mmucd); #015505-UCD) [39] were obtained from the Mutant Mouse Regional Resource Centers (NIH) and implanted and bred in-house at Thomas Jefferson University in a pathogen-free animal facility. Control C57BL/6 mice were obtained from the Frederick National Laboratory for Cancer Research (NCI).

### Antibodies

The following antibodies where purchased from BD Biosciences, unless otherwise noted, and used for flow cytometry staining: APC-Cy7-B220 (clone RA3-6B2), PerCP-CY5.5-CXCR5 (clone 2G8), APC-CD138 (clone 281-2), PE-Cy7-CD95/Fas (clone Jo2), FITC-GL7, eFluor 450-CD4 (clone RM4–5, eBioscience), PE-PD1 (clone J43, eBioscience), Alexa Fluor 700-CD38 (clone 90, eBioscience), rat anti-mouse CD16/32 (FcBlock; Pharmingen).

### Immunization, pathogenic challenges, sample collection and ELISA and virus neutralizing antibody assays to measure anti-RABV antibody titers

Groups of 6- to 10-week-old female IL-21R−/− or wild-type C57BL/6 mice were inoculated intramuscularly (i.m.) with 10^3^ or 10^5^ focus forming units (ffu)/mouse with rRABV or an equivalent volume of PBS as controls. Five weeks post-immunization, mice were challenged i.m. with 10^5^ ffu/mouse with CVS-N2c and then observed for three weeks for clinical signs of rabies. Mice were euthanized at the onset of neurological symptoms. At various times post-immunization and challenge, blood was collected retro-orbitally. Three-fold serial dilutions of sera were tested by ELISA to determine RABV G-specific IgG antibodies as described [10] and reported as the reciprocal serial dilution. Data represented two independent experiments (N = 9–11 mice per group). To measure virus neutralizing antibody titers, the Rapid Fluorescent Foci Inhibition Test (RFFIT) was completed on pooled sera from two independent experiments as described previously [16], [40], [41].

### Immunization, sample collection and flow cytometry analyses of T and B cell subsets from lymph nodes and bone marrow

Groups of 6- to 10-week-old female IL-21R−/− or wild-type mice were inoculated i.m. with 10^3^ or 10^5^ ffu/mouse with rRABV or PBS/naive as controls. Draining lymph nodes and bone marrow cells were collected 7 and/or 14 days post-immunization. Single cell suspensions (10^6^ cells/sample) were incubated with rat anti-mouse CD16/32 (1 ug/10^6^ cells) in fluorescence-activated cell sorter (FACS) buffer (PBS supplemented with 2% fetal bovine serum) for 1 h on ice. Cells were washed twice with FACS buffer and incubated with fluorescently conjugated antibodies (0.2 ug/10^6^ cells) for 30 min at RT in the dark. Cells were subsequently washed 2 times with FACS buffer and fixed in 2% paraformaldehyde in PBS for 30 minutes. Flow cytometry was completed using FACScan (BD LSRII) and analyzed by FlowJo software. Data represents samples completed in duplicate (N = 3–5 mice) [16].

### Statistical analysis

Kaplan-Meier survival curves were analyzed by the log rank test; ^*^p = <0.05 indicates significant survivorship between two immunization groups [9], [10]. Statistical difference between two groups of data was compared using an unpaired, two-tailed t test and data is presented at the mean ± SEM; *p<0.05, **p = 0.01–0.001, ***p≤0.001 [9], [10].

## Results

### IL-21 is critical for primary but not secondary antibody responses to live RABV-based vaccination

**Figure 1:**
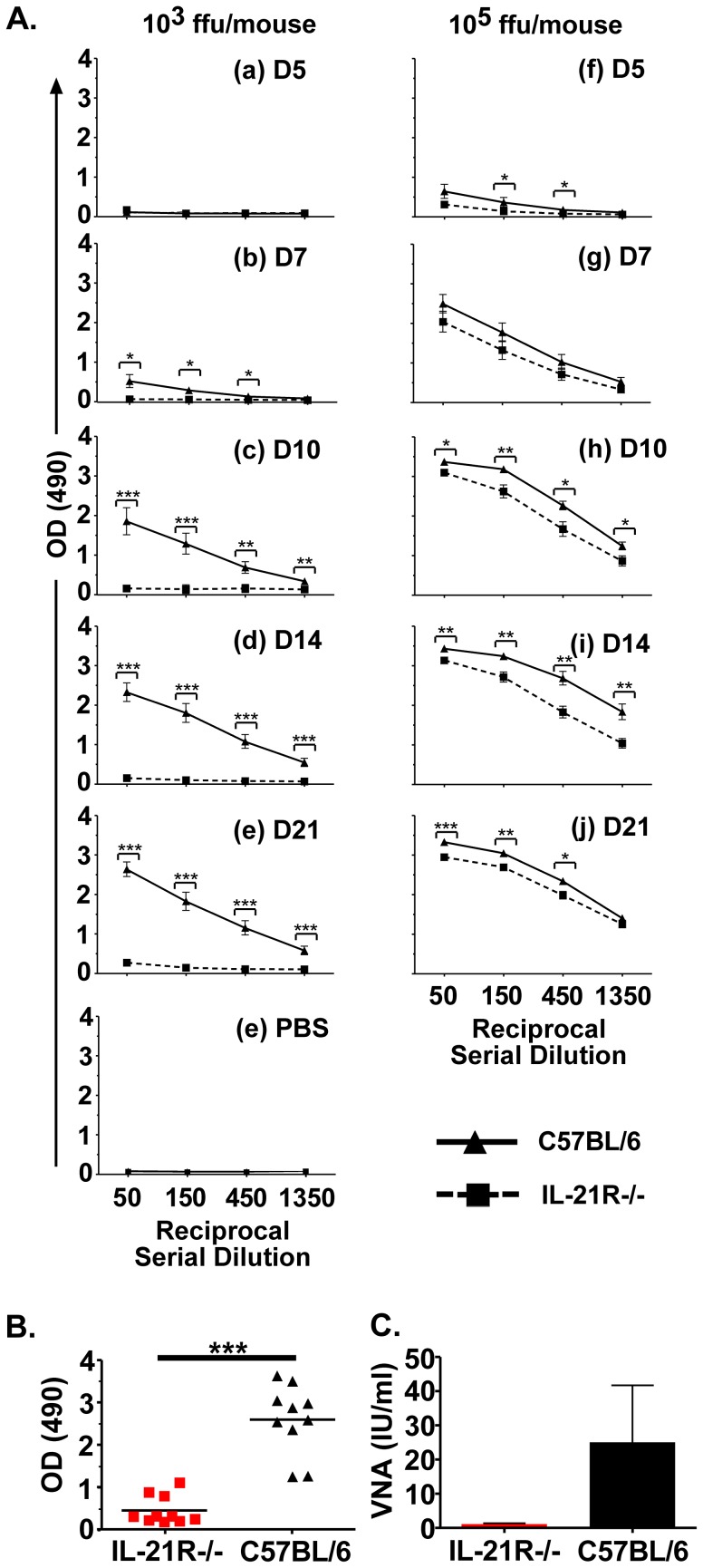
IL-21 signaling promotes optimal primary antibody responses induced by live RABV-based vaccination. A) IL-21R−/− or wild-type C57BL/6 mice were immunized i.m. with 10^3^ (left panels a–e) or 10^5^ (right panels f–j) ffu/mouse of rRABV and blood collected at the indicated time points. Four serial three-fold dilutions of sera were analyzed by ELISA to determine anti-RABV G antibody titers and presented as OD_490_ of the reciprocal serial dilution. For comparison, sera from PBS-immunized IL-21R−/− or wild-type mice (panel k) were tested in parallel and representative ELISA data for day 21 post-immunization is shown. B) Antibody titers for individual mice immunized with 10^3^ ffu/mouse [from Figure 1A(e)] are shown to indicate that at least three of the ten IL-21R−/− mice seroconverted against RABV G by day 28 post-immunization. C) VNA titers were measured from pooled sera collected 28 days post-immunization by RFFIT and expressed as International Units (IU/ml). The average of two independent experiments is shown. Statistical difference in antibody titers by ELISA between two groups of data was determined using an unpaired, two-tailed t test and data is presented at the mean ± SEM. *p<0.05, **p = 0.01–0.001, ***p≤0.001. (N = 9–11 mice per group from two independent experiments). (ffu = focus forming units; OD = optical density).

Cytokines present at the time of immunization have the ability to affect the outcome of vaccine-induced B cell responses. Due to the importance for IL-21 in promoting effective T-dependent B cell responses [21], [22], we examined the requirement for IL-21R signaling in the generation of antibodies in a mouse model of RABV immunogenicity and protection using a mouse model of IL-21R-deficiency. These mice, designated here as IL-21R−/− mice, lack the IL-21R extracellular and transmembrane domains but show normal lymphoid development [39]. T and B cells exhibit similar proliferative responses to CD3-speficic antibodies or LPS, respectively, when compared to wild-type controls [39]. As a group, IL-21R−/− mice immunized with a low dose of a live recombinant RABV-based vaccine (rRABV) (10^3^ ffu/mouse) showed only low levels of anti-RABV G antibodies that were not significantly different from PBS-immunized mice at all time points tested post-immunization (Figure 1, left panels, a–e), although at least three IL-21R−/− mice developed anti-RABV antibodies by day 28 post-immunization (Figure 1B). The seroconversion of these three IL-21−/− mice may explain the limited protection observed in the pathogenic challenge experiments described later in this report. Wild-type mice immunized with the same dose of rRABV showed significant levels of anti-RABV G antibodies as early as 7 days post-inoculation compared to rRABV-immunized IL-21R−/− mice, and these antibody responses continued to increase through day 21 post-immunization. VNA titers detected 28 days post-immunization (Figure 1C) are consistent with the antibody titers detected by ELISA (Figure 1B) indicating anti-RV antibody titers detected by ELISA are representative of the ability for vaccine-induced antibodies to neutralize rabies virus. IL-21R−/− mice immunized with a higher dose of rRABV (10^5^ ffu/mouse) showed significantly reduced anti-RABV antibody responses when compared to wild-type mice also immunized with 10^5^ ffu/mouse of rRABV (Figure 1, right panels, f–j). Together, this data indicates that IL-21 is critical for the induction of optimal primary anti-RABV antibody responses, especially when low doses of vaccine are used.

**Figure 2:**
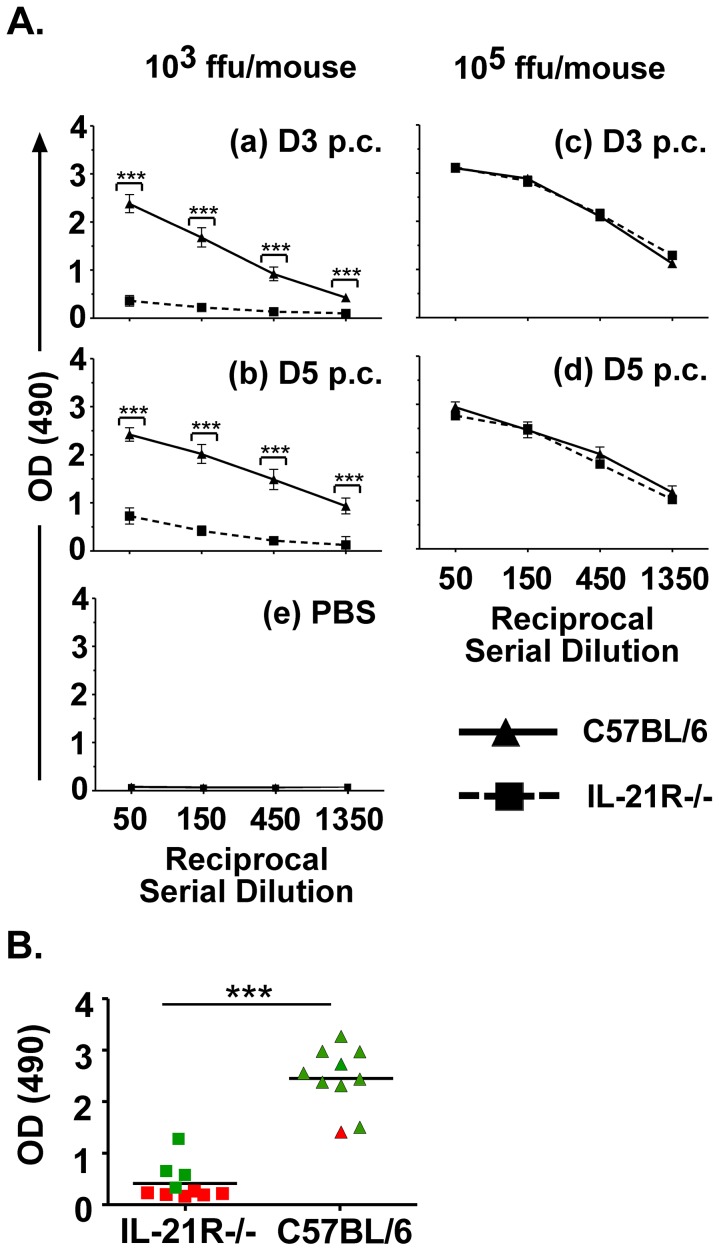
IL-21 signaling is dispensable for antibody recall responses to secondary RABV challenge. A) Five weeks post-immunization, mice immunized in Figure 1 with 10^3^ (left panels a and b) or 10^5^ (right panels c and d) ffu/mouse were challenged with 10^5^ ffu/mouse of pathogenic Challenge Virus Strain-N2c and sera analyzed 3 and 5 days post-challenge by ELISA for anti-RABV G antibody recall responses as described in Figure 1. PBS-immunized IL-21R−/− and wild-type mice were also tested in parallel and a representative ELISA data is shown in panel e. B) Antibody titers for individual mice immunized with 10^3^ ffu/mouse rRABV and then challenged [from Figure 2A(b)] are shown to indicate an antibody recall response against RABV G by day 3 post-challenge in a minimum number of IL-21R−/− mice. Mice that survived challenged are shown with a green icon, mice that did not survive challenge are shown with a red icon. Statistical difference in antibody titers by ELISA between two groups of data was determined using an unpaired, two-tailed t test and data is presented at the mean ± SEM. *p<0.05, **p = 0.01–0.001, ***p≤0.001. (N = 9–11 mice per group from two independent experiments). (p.c. = post-challenge; ffu = focus forming units; OD = optical density).

**Figure 3:**
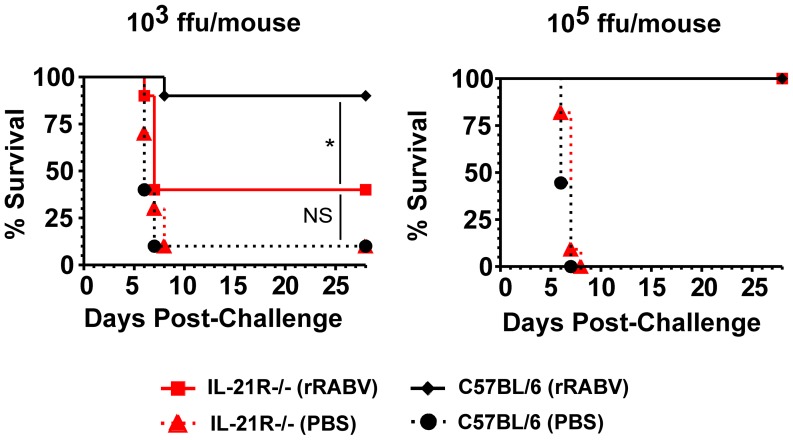
IL-21 signaling is important for protection against RABV challenge after low-dose vaccination. Immunized (Figure 1) and challenged (Figure 2) mice were monitored daily for 28 days post-challenge for clinical signs of rabies and euthanized at the first neurological symptoms of RABV infection (10^3^ ffu/mouse left panel; 10^5^ ffu/mouse, right panel). Kaplan-Meier survival curves were analyzed by the log rank test; *p<0.05. (N = 9–11 mice per group from two independent experiments). (ffu = focus forming units).

We next wanted to evaluate the effect of IL-21 on vaccine-induced antibody recall responses and protection against pathogenic RABV challenge. Five weeks post-immunization with rRABV, mice from Figure 1 were challenged with 10^5^ ffu/mouse of a highly pathogenic mouse-adapted RABV strain (Challenge Virus Strain-N2c; CVS-N2c) [38], which typically kills naïve mice within 8 days post-infection [9], [10]. Consistent with the low antibody titers detected during the primary antibody response, significantly less anti-RABV antibodies were detected three or five days post-challenge in IL21R−/− mice immunized with 10^3^ ffu/mouse of rRABV compared to the antibody recall response detected in wild-type mice (Figure 2, panels a and b, and Figure 2B) and antibody recall titers were not significantly different from PBS-immunized/CVS-N2c-challenged mice (Figure 2, e). Only 40% of IL-21R−/− mice immunized with 10^3^ ffu/mouse of rRABV were protected against pathogenic RABV challenge, while all wild-type mice that were similarly immunized were protected against challenge (Figure 3, left panel). As expected, those mice with higher antibody titers showed protection compared to mice with lower antibody titers (Figure 2B). Conversely, an antibody recall response was induced in IL-21R−/− mice immunized with 10^5^ ffu/mouse of rRABV within three days post-challenge with CVS-N2c at levels equivalent to rRABV-immunized wild-type mice (Figure 2, panels c and d) and all immunized IL-21R−/− mice were protected against pathogenic RABV challenge (Figure 3, right panel). Taken together, IL-21 is required for optimal primary (Figure 1) but not secondary (Figure 2) antibody responses to RABV vaccination. Furthermore, IL-21 is required for protection against pathogenic RABV in a vaccine dose-dependent manner (Figure 3).

### IL-21 is dispensable for T_fh_ formation at all vaccine doses tested but required for optimal GC B cells in a vaccine dose-dependent manner

**Figure 4:**
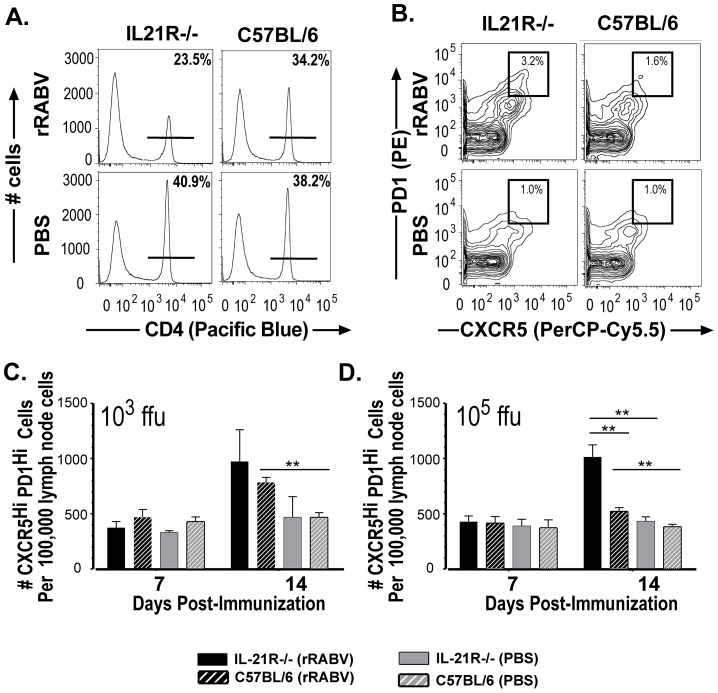
IL-21 is dispensable for T_fh_ cell development in response to rRABV-based vaccination. IL-21R−/− or C57BL/6 mice were immunized i.m. with a single dose of 10^3^ or 10^5^ ffu/mouse with rRABV and T_fh_ cell development was analyzed at the indicated time points post-immunization. A, Representative gating strategy from IL-21R−/− or C57BL/6 mice immunized with rRABV or an equal volume of PBS as a control to identify CD4^+^ T cells from total live draining lymph node cells. B, Representative gating strategy from IL-21R−/− or wild-type mice immunized with rRABV or PBS to identify T_fh_ (CD4^+^CXCR5^hi^PD1^hi^) cells from the CD4^+^ T cell population. C and D, Percentage of T_fh_ cells in the total CD4^+^ T cell population in lymph nodes was determined in IL-21R−/− or C57BL/6 mice 7 and 14 days post-immunization with 10^3^ (C) or 10^5^ (D) ffu/mouse rRABV or PBS. Statistical difference in T cell data between two groups of data was determined using an unpaired, two-tailed t test and data is presented at the mean ± SEM. *p<0.05, **p = 0.01–0.001, ***p≤0.001; (N = 5 mice per group).

**Figure 5:**
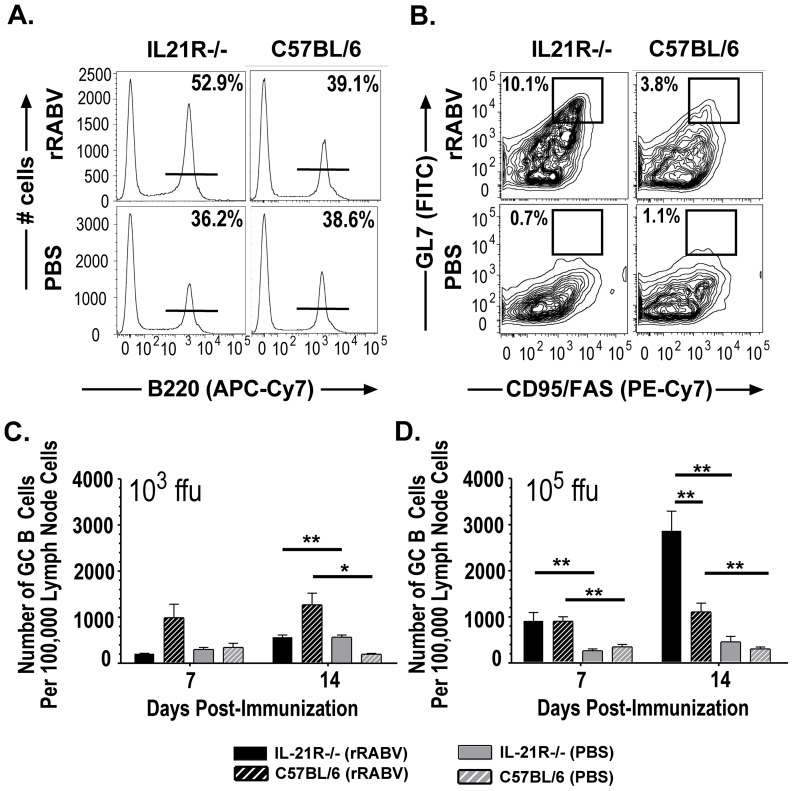
IL-21 promotes optimal GC B cell development in a vaccine dose-dependent manner. Draining lymph node cells from IL-21R−/− or wild-type C57BL/6 mice immunized in Figure 4 were analyzed for the presence of B cells displaying a GC phenotype (B220^+^GL7^hi^CD95/Fas^hi^). A, Representative gating strategy from IL-21R−/− or C57BL/6 mice immunized with rRABV or PBS to identify B220^+^ B cells from total live lymph node cells. B, Representative gating strategy from IL-21R−/− or wild-type mice immunized with rRABV or PBS to identify GC B cells. C and D, Number of GC B cells per 100,000 draining lymph node cells was determined in IL-21R−/− or wild-type mice immunized with 10^3^ (C) or 10^5^ (D) ffu/mouse 7 or 14 days post-immunization with rRABV or PBS alone. Statistical difference in GC B cell data between two groups of data was determined using an unpaired, two-tailed t test and data is presented at the mean ± SEM. *p<0.05, **p = 0.01–0.001, ***p≤0.001; (N = 5 mice per group); (ffu = focus forming units).

Next we wanted to determine whether the impaired primary anti-RABV antibody response in IL-21R−/− mice was due to an overall defect in the generation of T_fh_ and/or GC B cells. Lymph nodes from IL-21R−/− or wild-type mice were collected 7 or 14 days post-immunization with 10^3^ or 10^5^ ffu/mouse of rRABV or PBS alone to determine the influence of IL-21 on T_fh_ and B cell populations. Representative gating strategies [17], [42] to identify CD4^+^ T cells from the total live lymph node cultures (Figure 4A) or T_fh_ (CD4^+^CXCR5^hi^PD1^hi^) cells from the CD4^+^ T cell populations (Figure 4B) are shown. A significant increase in the number of CD4^+^ T cells displaying a T_fh_ phenotype was detected in IL-21R−/− mice 14 days post-immunization with 10^3^ or 10^5^ ffu/mouse rRABV compared to similarly immunized wild-type mice (Figure 4C and Figure 4D, respectively). However, the formation of optimal GC B cells appears to be dependent on the dose of vaccine administered (Figure 5). Representative gating strategies [22], [43], [44] to identify B220^+^ B cells from the total live lymph node cultures (Figure 5A) or GC B cells (B220^+^GL7^hi^CD95/Fas^hi^) from the B220^+^ B cell population (Figure 5B) are shown. IL-21R−/− mice immunized with a low dose of vaccine failed to induce optimal GC B cell formation compared to wild-type mice 14 days post-immunization, as shown by a significant decrease in the number of GC B cells in IL-21R−/− mice compared to wild-type mice (Figure 5C). However, a significant increase in the number of GC B cells was detected in IL-21R−/− mice immunized with 10^5^ ffu/mouse of rRABV compared to wild-type mice 14 days post-immunization (Figure 5D). The data indicates that the suboptimal primary antibody responses detected in IL-21R−/− mice immunized with 10^3^ ffu/mouse of rRABV appears to be due to the lack of GC B cell formation, while the suboptimal primary antibody response detected in IL-21R−/− mice immunized with 10^5^ ffu/mouse most likely does not result from a defect in T_fh_ or GC B cell development.

### IL-21 is dispensable for memory B cells formation but required for plasma cell generation in response to live RABV-based vaccination

**Figure 6:**
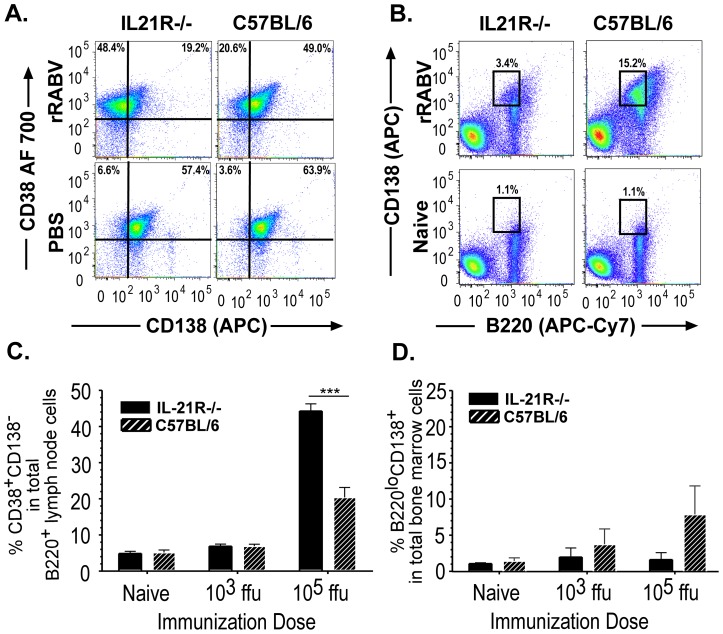
IL-21 is dispensable for memory B cells but required for optimal PC formation. IL-21R−/− or C57BL/6 mice were immunized i.m. with a single dose of 10^3^ or 10^5^ ffu/mouse with rRABV and memory B cell or PC populations were analyzed 7 or 14 days post-immunization, respectively. A, B220^+^ B cells were gated from the total live draining lymph node cell population as described in Figure 5. Representative gating strategy from IL-21R−/− or C57BL/6 mice immunized with rRABV or PBS to determine memory B cells (B220^+^CD38^+^CD138^−^) from total B220^+^ B cells in the draining lymph node. B, Representative gating strategy from IL-21R−/− or C57BL/6 mice immunized with rRABV or PBS to identify PCs (B220^lo^CD138^+^) from the total cells in the bone marrow. C, Percentage of B cells displaying a memory B cell phenotype in the lymph node was determined in IL-21R−/− or C57BL/6 mice immunized with 10^3^ or 10^5^ ffu/mouse at 7 days post-immunization with rRABV or PBS. D, Percentage of PCs in the BM cell population was determined from IL-21R−/− or C57BL/6 mice immunized with 10^3^ or 10^5^ ffu/mouse at 14 days post-immunization with rRABV or PBS. Statistical difference in B cell data between two groups of data was determined using an unpaired, two-tailed t test and data is presented at the mean ± SEM. *p<0.05, **p = 0.01–0.001, ***p≤0.001; (N = 5 mice per group).

Our analysis above shows that IL-21R signaling is dispensable for the formation of the T_fh_ and GC B cell populations in response to higher doses of rRABV, indicating that other B cell types are more likely responsible for the suboptimal primary antibody titers detected in IL-21R−/− mice immunized with 10^5^ ffu/mouse. Since IL-21 can also influence the balance between the generation of memory B cells and PCs [24], [45]–[47], we investigated the role for IL-21R signaling to regulate memory B cell and PCs populations in response to RABV vaccination. Figure 6A and Figure 6B show representative gating strategies [48]–[50] to identify the memory B220^+^ B cells (CD38^+^CD138^−^) from the lymph node and PC (B220^lo^CD138^+^) populations from the bone marrow from mice immunized with 10^3^ or 10^5^ ffu/mouse of rRABV or PBS alone. The presence or absence of IL-21R does not appear to influence the development of memory B cells in mice immunized with 10^3^ ffu/mouse of rRABV (Figure 6C). However, the percentage of memory B cells was significantly increased in the lymph node cell cultures from IL-21R−/− mice immunized with 10^5^ ffu/mouse of rRABV as early as 7 days post-immunization compared to immunized wild-type mice (Figure 6C). By day 14 post-immunization, similar memory B cell populations were measured (data not shown), indicating that IL-21 is not required for the formation of memory B cells in response to live RABV-based vaccination. This is consistent with the findings in Figure 1 and Figure 2 indicating that IL-21 is dispensable for secondary antibody responses against RABV infection when a primary antibody response develops. However, the percentage of PCs was reduced in the bone marrow of IL-21R−/− mice immunized with 10^5^ ffu/mouse compared to rRABV- or PBS-immunized wild-type mice 14 days post-immunization (Figure 6D), consistent with the suboptimal primary antibody titers detected in IL-21R−/− mice.

## Discussion

Current rabies PEP regimens are based on multiple doses of inactivated RABV-based vaccines administered intramuscularly or intradermally. In cases of severe exposure, rabies immune globulin (RIG) is administered [51]–[53]. The development of a single-dose vaccine would greatly benefit human rabies prevention by reducing the cost of vaccination and saving lives. Understanding immune parameters that influence the magnitude and/or quality of anti-RABV antibody responses may lead to more effective single-dose vaccines [54]. Correlates of protection against rabies infections are defined as virus neutralizing antibodies directed against the single viral transmembrane glycoprotein (G) [15], [52], [54], [55]. CD8^+^ T cells do not appear to be important for the clearance of RABV infections [56]. Protection against RABV infection typically requires CD4^+^ T cell help [56]–[60], although we recently showed that this requirement is not absolute and that protection against pathogenic RABV challenge can be afforded in mice devoid of all T cells (TCRβδ−/− mice) vaccinated with a matrix gene-deleted RABV-based vaccine (rRABV-ΔM) [16]. Furthermore, we show that mice immunized with rRABV-ΔM also induce antibodies by T cell-dependent extrafollicular B cell responses before GC-derived B cells are detected. Together, our previous work identified multiple pathways of B cell development that can be exploited to make more efficacious RABV-based vaccines for use in humans. Nonetheless, very little information is available on how effective B cells develop in response to live RABV-based vaccination. IL-21 is a pleotropic cytokine that is produced by NKT cells and CD4^+^ T cells, most notably Th17 and T_fh_ cells. IL-21 binds to the IL-21R on a wide variety of cells involved in innate immunity, including DCs, NK cells, NKT cells, and macrophages, as well as on cells involved in adaptive immunity, such as B cells and CD4^+^ or CD8^+^ T cells [reviewed in [20]]. Due to its multiple roles in innate and adaptive immunity, IL-21 has the potential to influence the quality and magnitude of vaccine-induced immunity to acute viral infections. Here we used a mouse model of IL-21R-deficiency to evaluate the role for IL-21R signaling in vaccine-induced protection against RABV; i.e., an acute viral infection that relies on B cells for protection that has implications for global public health initiatives.

In this report, we showed that IL-21R signaling is critical for the generation of optimal primary anti-RABV antibody responses to vaccination. Primary anti-RABV antibody titers were significantly reduced in immunized IL-21R−/− mice compared to wild-type mice at almost all time points tested post-immunization, suggesting IL-21R signaling plays important roles throughout RABV-specific primary B cell responses. Nonetheless, IL-21R signaling appears to influence immunity in a vaccine dose-dependent manner, which is consistent with findings by others suggesting the influence of IL-21 is dependent on the model studied [22], [61]. Indeed, significantly less IL-21R−/− mice immunized with low-dose vaccination were protected against pathogenic challenge compared to wild-type mice while all IL-21R−/− and wild-type mice immunized with high-doses of vaccine survived challenge similarly. Despite the differences in protection elicited in IL-21R−/− mice immunized with different doses of vaccine, it appears that IL-21 is critical for the generation of optimal RABV-specific primary B cell responses.

One potential explanation for the suboptimal primary antibody responses observed in immunized IL-21R−/− mice compared to immunized wild-type mice might be that GC B cells failed to form in IL-21R−/− mice, therefore, the GC B cell compartment was analyzed in mice immunized with different doses of vaccine. GC-derived B cells were reduced in IL-21R−/− mice immunized with a low dose of vaccine compared to similarly immunized wild-type mice, indicating that IL-21 is required for optimal GC B cell formation in response to low-dose RABV-based vaccination. On the other hand, GC-derived B cells expanded in IL-21R−/− mice immunized with a high dose of vaccine compared to similarly immunized wild-type mice, indicating that IL-21 is dispensable for GC B cell formation after high-dose vaccination with rRABV-based vaccines. Furthermore, the data indicates that factors other than IL-21 were responsible for GC B cell formation in IL21R−/− mice immunized with higher doses of vaccine. Multiple signals lead to B cell activation and functions. These signals can come from BCR or Toll-like receptor (TLR) ligation, TNF superfamily receptor engagement (eg., via BAFF and APRIL) or cytokine signaling. Furthermore, B cell activation is contextual, meaning B cells are differentially activated in the presence of different signals at the time of antigen exposure. Due to the repetitive display of rabies antigen on the surface of infectious particles, the potential exists that cross-linking BCRs and/or TLRs on the surface of B cells overcame the requirement for IL-21R signaling in B cell activation when high doses of vaccine are administered. The influences of these and other B cell signaling events in the context of RABV-based vaccine-induced B cell activation were not directly measured in these studies and remain to be elucidated. Nonetheless, based on the results reported here, it appears that IL-21R signaling is important for optimal primary vaccine-induced antibody responses to RABV vaccination especially when low doses of vaccine are administered.

As noted above, a rapid antibody response is critical for rabies PEP to neutralize virus before it reaches the CNS. We have recently shown that RABV-based vaccines are able to induce early and rapid T cell-dependent extrafollicular antibody responses before GC B cells are formed [16]. These early pre-GC B cell responses contributed to the protection against pathogenic RV challenge early post-immunization, which is an important attribute for PEP [16]. In the studies described in this report, we detected a significant reduction in antibody titers in IL-21R−/− mice as early as 5 days post-immunization with a high dose of rRABV compared to immunized wild-type mice, suggesting that IL-21 may be influencing the outcome of extrafollicular antibody responses in the context of RABV vaccination, although this was not directly studied in this report. Nonetheless, the frequency of T_fh_ and GC B cells was similar in IL-21R−/− mice compared to wild-type mice 7 days post-immunization and, therefore, it would appear that the early suboptimal antibody responses in IL-21R−/− mice may be due to impaired extrafollicular PCs directly and not through impaired T_fh_ or GC B cell formation. This is consistent with the findings that IL-21 can promote Blimp-1 expression and PC development [47], IL-21- or IL-21R-deficiency decreases extrafollicular PCs in a model of NP-KLH immunity [62], and that IL-21 acts on early stages of B cell differentiation before GC or PC B cells are formed [42]. Finally, IL-21 has been reported to be important for T_fh_ cell maintenance but not formation. Together, existing data suggests that IL-21R−/− signaling influences early events in pre-GC B cell development in the context of RABV vaccination [22].

IL-21 can also influence the balance of B cell differentiation into memory B cells or PCs [45]–[47]. The specific role for IL-21 in memory B cell responses is not completely clear and appears to rely on the type of antigen used and the model studied [61], [62]. In the context of RABV vaccination, IL-21R signaling was not required for the generation of B cells displaying a memory B cell phenotype in IL-21R−/− mice immunized with either vaccine dose. This is consistent with our finding showing that IL-21R signaling is not required for optimal secondary anti-RABV G antibody titers after challenge with pathogenic RABV. However, we detected a decrease in the number of PCs in IL-21R−/− mice immunized with either dose of vaccine compared to wild-type mice. We cannot determine whether the slightly suboptimal PC subset detected in IL-21R−/− mice immunized with low doses of vaccine was indirectly a result of impaired GC B cell development or directly as a result of impaired PC formation itself. However, in mice immunized with a high dose of vaccine where we observed an expansion of GC-derived B cells in IL-21R−/− mice, we also observed a decrease in PCs in the bone marrow compared to wild-type mice, indicating that IL-21 acts directly on the formation of PCs. Together, the data shows that IL-21 influences the balance between memory and PC B cell formation in the context of RABV vaccination.

Despite the impaired primary antibody response and PC B cell formation in immunized IL-21R−/− mice, we detected an expansion of CD4^+^ T cells displaying a pre-T_fh_ (data not shown), T_fh_ cell and GC B cell phenotype in IL-21R−/− mice compared to wild-type mice at 14 days post-immunization. The expansion of GC B cells and T_fh_ cells in the absence of IL-21R signaling was also shown by King I.L. et al in a model of *Heligmosomoides polygyrus* immunity [24], suggesting that IL-21R signaling may not be necessary for the generation of these cell types in response to a wide range of pathogens or vaccination. Furthermore, the increase in GC B cells and T_fh_ cells in *H. polygyrus*-infected or RABV-vaccinated IL-21R−/− mice suggests that IL-21R signaling may play an inhibitory role in the development of T and B cells in the context of some pathogens, which is consistent with the ability for IL-21 to activate or inhibit immune function depending on the antigen and available co-stimulatory signals [20]. The expansion of T_fh_ and GC B cells in IL-21R−/− mice compared to wild type mice is also consistent with the finding that IL-21 has the ability to mediate apoptosis in primary resting and activated murine B or to promote apoptosis or growth arrest for non-specifically activated B cells [63]. Alternatively, the elevated number of GC T_fh_ cells could be a result of the lack of PC that developed in the IL21R−/− mice. Pelletier et al described a negative regulatory feedback-loop in which antigen-specific PCs negatively regulate antigen-specific T_fh_ cell development and function [64]. In this report, they also observed a significant expansion of T_fh_ cells and GC B cells in the absence of PC development. Together, the role for IL-21 in the homeostatic balance of T and B cell development in the context of infectious diseases appears to be important and remains to be fully elucidated.

Additional studies are needed to identify the exact cell type(s) responsible for the affects described in this report. While we speculate that B cell-intrinsic IL-21R signaling is responsible for the induction of optimal anti-RABV antibody responses, we cannot rule out the influence of other cell types that also express IL-21R. IL-21 has the ability to influence the function of macrophages, NK cells and NKT cells by affecting survival/apoptosis, antigen processing, and cytokine secretion [reviewed in [20]]. The function of these cells of the innate immune system may indirectly be affecting the outcome of B or T cell functions in the context of RV vaccination. Nonetheless, IL-21 has the potential to influence a wide range of B cell functions and pathways. Our preliminary data indicates that IL-21 is critical for the formation of optimal vaccine-induced primary antibody responses and demonstrates an important role for IL-21 in the generation of vaccine-induced immunity against RABV infection and perhaps other acute infections that rely on B cell-mediated effector functions for protection.
